# Factors related to late stage diagnosis of oral squamous cell carcinoma

**DOI:** 10.4317/medoral.17399

**Published:** 2011-07-15

**Authors:** Juan-Manuel Seoane-Romero, Inés Vázquez-Mahía, Juan Seoane, Pablo Varela-Centelles, Inmaculada Tomás, José-Luis López-Cedrún

**Affiliations:** 1 Stomatology Department. School of Medicine and Dentistry. University of Santiago de Compostela. Spain; 2 Oral and Maxillofacial Surgery. CHUAC. A Coruña. Spain

## Abstract

Aims: To identify factors related to advanced-stage diagnosis of oral cancer to disclose high-risk groups and facilitate early detection strategies. Study design: An ambispective cohort study on 88 consecutive patients treated from January 1998 to December 2003. Inclusion criteria: pathological diagnosis of OSCC (primary tumour) at any oral site and suffering from a tumour at any TNM stage. Variables considered: age, gender, smoking history, alcohol usage, tumour site, macroscopic pattern of the lesion, co-existing precancerous lesion, degree of differentiation, diagnostic delay and TNM stage. Results: A total of 88 patients (mean age 60±11.3; 65.9% males) entered the study. Most patients (54.5%) suffered no delayed diagnosis and 45.5% of the carcinomas were diagnosed at early stages (I-II). The most frequent clinical lesions were ulcers (70.5%). Most cases were well- and moderately-differentiated (91%). Univariate analyses revealed strong associations between advanced stages and moderate-poor differentiation (OR=4.2; 95%CI=1.6-10.9) or tumour site (floor of the mouth (OR=3.6; 95%CI=1.2-11.1); gingivae (OR=8.8; 95%CI=2.0-38.2); and retromolar trigone (OR=8.8; 95%CI=1.5-49.1)). Regression analysis recognised the site of the tumour and the degree of differentiation as significantly associated to high risk of late-stage diagnosis. Conclusions: Screening programmes designed to detect asymptomatic oral cancers should be prioritized. Educational interventions on the population and on the professionals should include a sound knowledge of the disease presentation, specifically on sites like floor of the mouth, gingivae and retromolar trigone. More studies are needed in order to analyse the part of tumour biology on the extension of the disease at the time of diagnosis.

** Key words:** Oral cancer, advanced-stage, diagnosis, cohort study.

## Introduction

Survival rates for oral cancer are very poor (around 50% overall), and no remarkable improvements have occurred in recent decades despite advances in therapeutic interventions (1). Variables like age, co-morbidity, immunological or nutritional status, size and location of the tumour, nodal status, oncogene expression, proliferation markers, or DNA content have been assessed as independent prognostic markers for oral cancer (2), but stage at diagnosis remains as the most important prognostic indicator for oral and oropharyngeal squamous cell cancers (SCCs) in such a way that advanced stages are frequently associated with high mortality rates (3-5).

Advances in therapy and standards of care are likely to have played a role in the moderate increase of survival trends, particularly for females and tongue cancer (6,7).

Detecting oral cancer at an early stage is believed to be the most effective means of reducing rates of death, morbidity and disfigurement from this disease (1), but progression in this field is slow: late-stage presentation is commonplace despite the existing evidence supporting that visual and tactile exploration may ease detection of oral cancer at early stages (8-10). Evidence also suggests that an oral examination of high risk individuals may be a cost-effective screening strategy (11).

An important number of studies have assessed the determinants for diagnostic delay (period elapsed since the first sign or symptom until definitive diagnosis) despite its controversial part in oral cancer (12-14), but the reports aimed at identifying predictors for diagnosis at advanced stages are very scarce though tumour stage is directly related to mortality by oral cancer.

This study was designed to analyse the hypothetical factors related to diagnosis of oral cancer at advanced stages (III-IV) in order to identify high-risk groups for late-stage diagnosis and facilitate early detection strategies.

## Material and Methods

An ambispective cohort study was designed to analyse those factors related to late-stage oral cancer diagnosis. The study sample was made of 88 patients treated at the Oral and Maxillofacial Surgery Service of the CHUAC from January 1998 to December 2003 that met the following inclusion criteria: pathological diagnosis of OSCC (primary tumour) at any oral site and suffering from a tumour at any TNM stage.

The primary sites of oral cancer were: buccal mucosa (n=5), upper and lower gingiva (n=15), hard palate (n=2), tongue (n=32), floor of the mouth (n=24) and retromolar trigone (n=10).

The variables considered included age, gender, smoking history, alcohol usage, tumour site, macroscopic pattern of the lesion (ulcerated, exophytic or mixed), coexisting precancerous lesion, and degree of differentiation.

The time interval from the self-reported date when oral cancer signs and/or symptoms were first noted by the patient to the date of definitive pathological diagnosis was defined as the total diagnostic delay. In order to limit the recall bias inherent to this kind of studies, delay data collected from the patient was also validated by those obtained from close relatives. In both situations, identical structured interviews were undertaken for all cases. The median of total diagnostic times has been used as a cut-off point to distinguish between delayed and non-delayed cases in a more objective way.

TNM stage was considered as the dependent variable (early = tumour-node-metastasis [TNM] stage I or II; advanced = TNM stage III or IV). Early stages include a variety of tumour sizes (<4 cm) without invasion of adjacent structures, and no lymph node or distant metastases. Advanced stages include tumours invading adjacent structures, e.g., through cortical bone, into deep (extrinsic) muscle of tongue, maxillary sinus, and skin, or a more advanced node status than early stages’ or display distant metastases (15). 

 -Statistical analysis

Data were entered on the PASW statics18 statistical package and the sample characterized by the variables of interest. A descriptive study was conducted where quantitative variables were expressed as mean ± standard deviation; and qualitative ones as absolute frequency and percentage. 

Means were compared using the Student’s t test after assessing their normality with the Kolmogorov-Smirnov test. Those variables that are clinically relevant or were significantly related to advanced TNM-stage after univariate analysis (simple logistic regression) were included in a multivariate model (multiple logistic regression). The significance level chosen for all tests was p<0.05.

## Results

A total of 88 patients (mean age 60±11.3), mostly males (65.9%) entered the study. The most frequent tumour sites were tongue (36.4%), floor of the mouth (27.3%) and gingivae (17%).

The median for the interval between the first sign/symptom to pathological diagnosis was 45 days, and most patients (54.5%) suffered no delayed diagnosis. A 45.5% of the oral carcinomas were diagnosed at early stages (I-II). The most frequent clinical lesions were ulcers (70.5%), being the cancer associated to a precancerous lesion in a 16.5% of the cases. 

Most cases were well- and moderately-differentiated (91%) ( Table 1). 

Univariate analyses revealed that age (OR=1.0; 95%CI=0.9-1.0), smoking habit (OR=1.4; 95%CI=0.5- 3.9), alcohol usage (OR=1.0; 95%CI=0.4-2.6 ), co-existence of a precancerous lesion (OR=0.6; 95%CI=0.2-2.1) and the clinical presentation (ulcerated/mixed) of the oral carcinoma (OR=2.7; 95%CI=0.7-9.9) were neither significantly associated to diagnosis at advanced-stages, nor to TNM-advanced stage (OR=0.7; 95%CI=0.3-1.6).

On the other hand, male gender was identified as a risk factor for late TNM stage at diagnosis (OR=3.8; 95%CI=1.4-9.6). Strong associations between advanced stages and moderate-poor differentiation (OR=4.2; 95%CI=1.6-10.9) or tumour site (floor of the mouth (OR=3.6; 95%CI=1.2-11.1); gingivae (OR=8.8; 95%CI=2.0-38.2); and retromolar trigone (OR=8.8; 95%CI=1.5-49.1)) have also been identified by univariate analysis ( Table 2).

Regression analysis excluded “gender” from the multivariate model, remaining tumour site and degree of differentiation signifi-cantly associated to high risk of late-stage diagnosis ( Table 3).

**Table 1:**
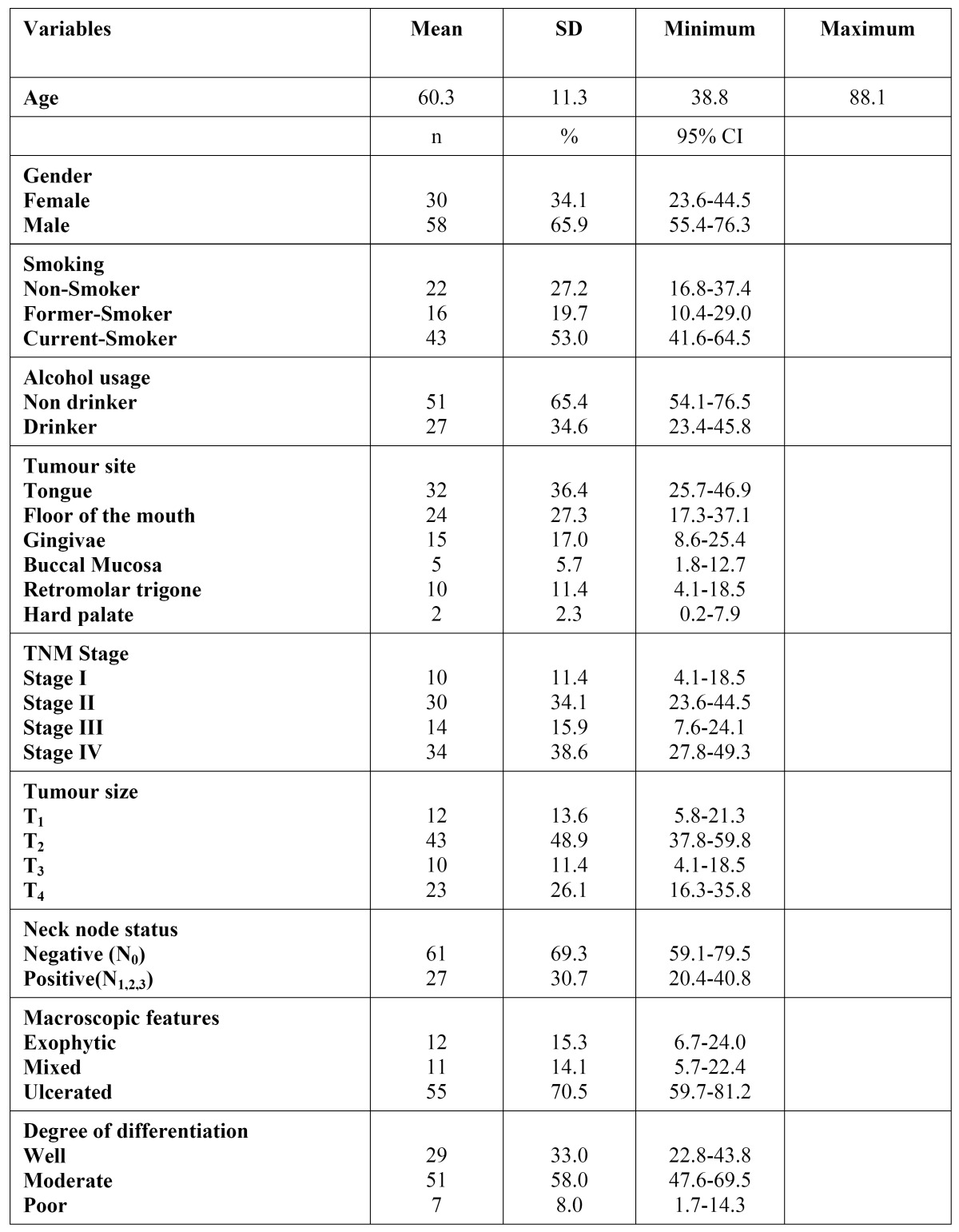
Description of the sample (n=88) .

**Table 2:**
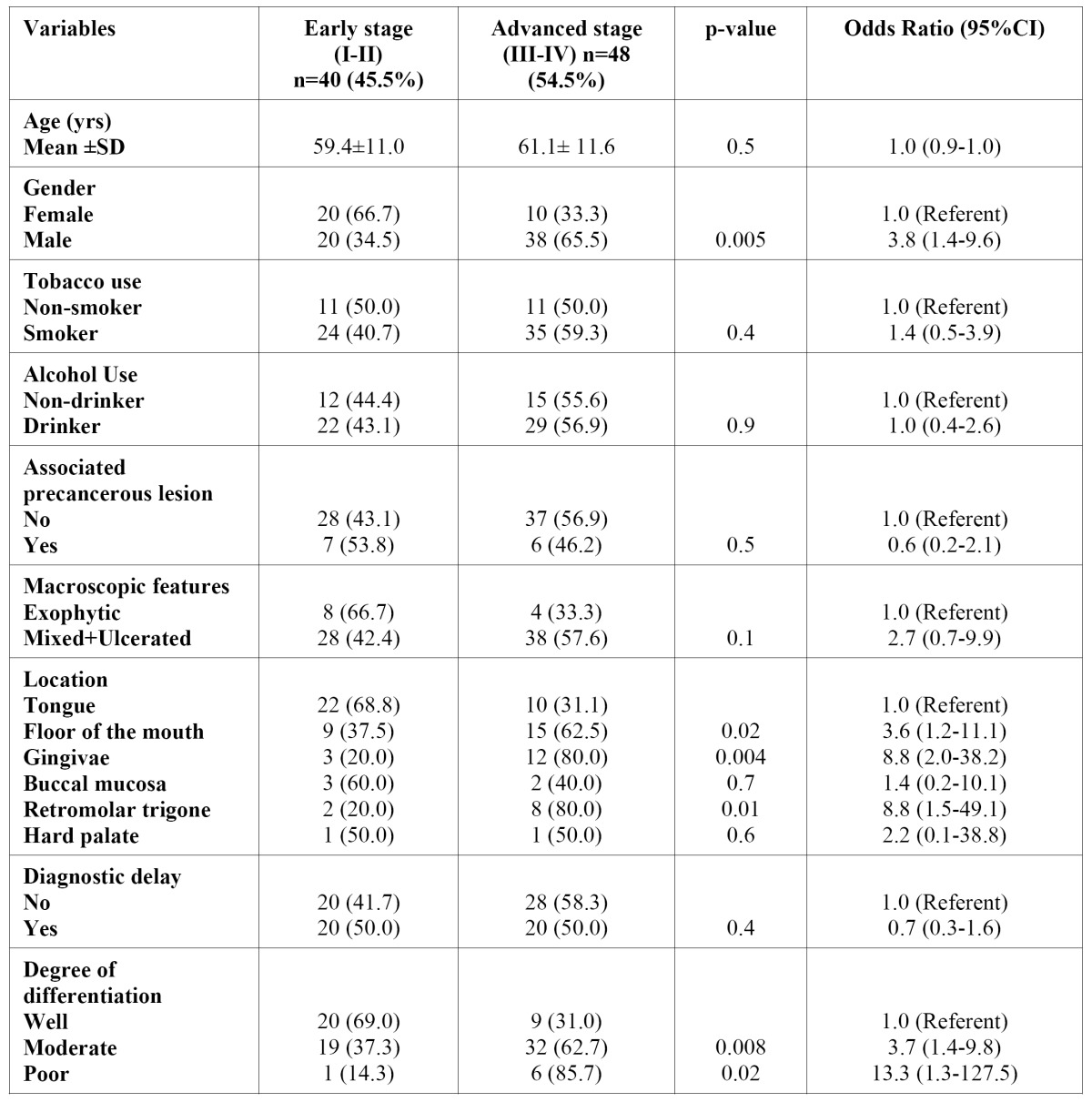
Patient characteristics distribution according to TNM-stage at diagnosis. Simple logistic regression analysis.

**Table 3:**
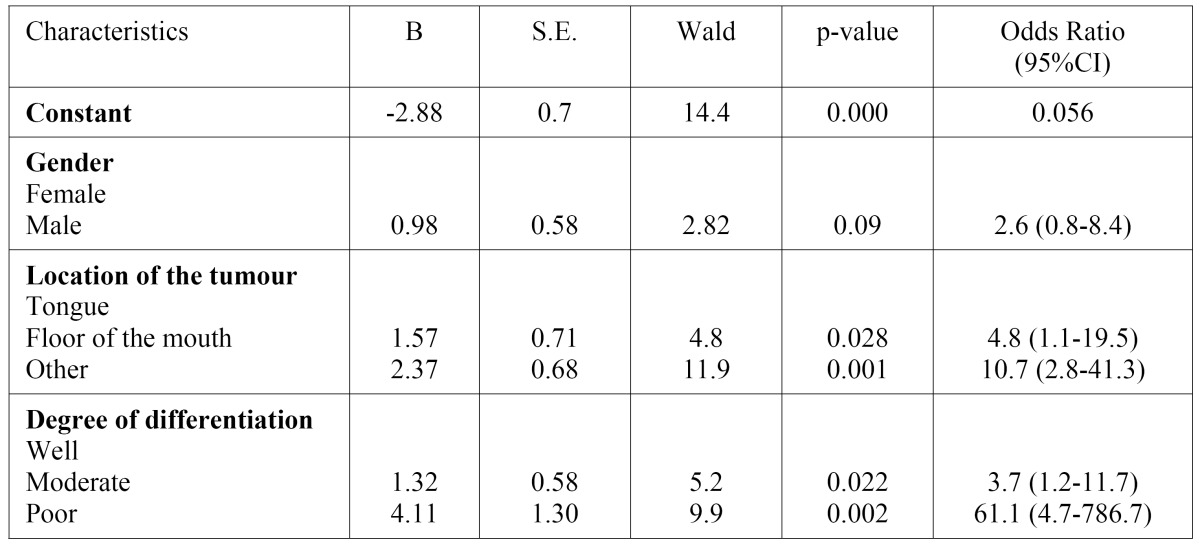
Multiple logistic regression analysis of the association between advanced staged and patients/tumours characteristics.

## Discussion

The current recommendations to screen for oral cancer at every routine check-up is not practical and has not produced the intended results. Selective opportunistic screening may be a more realistic and effective solution. Detection of oral and oropharyngeal SCCs during a non-symptom-driven examination has proved an association to lower stage at diagnosis, in the same way as patients with a regular primary care dentist are significantly more likely to be diagnosed at early stages (4,16).

Unfortunately, about a 60% of cancers are identified late (stages III or IV) with survival rates ranging from 10% to 40% after 5 years (17,18). Up to a 54.5% of the patients in this series were diagnosed at late stages, and recognition of predictors for advanced-stage diagnosis could permit the development of strategies aimed at improving this percentage.

Age, gender, and tobacco and alcohol consumption did not behaved as variables linked to late-stage diagnosis; as were not previously associated to professional or patient-related diagnostic delays (19,20). The existence of precancerous lesions associated to the tumour did not seem to modify the extension of the disease at the moment of diagnosis, despite that proliferative verrucous leukoplakia or the presence of mild or moderate epithelial dysplasia at the margins of a surgically removed OSCC carries a significant risk of local recurrence and modifying prognosis (21).

Ulcerated-type OSCC were diagnosed mostly (up to a 60%) at stages III-IV, but this association did not reach statistical signification. Moreover, the predictive value for survival of the lesion clinical appearance is controversial, although it is accepted that ulcerated lesions imply poorer survival rates (22).

Previous reports have described the association between primary tumour site and delayed diagnosis or diagnosis at advanced stages (23): tongue, buccal mucosa and lip have been recognised as locations that favour early-stage diagnosis (18), whereas the floor of the mouth and the retromolar trigone have been linked to diagnosis at advanced stages; locations like palate or gingivae showed contradictory results (18,24). Our data show that the floor of the mouth, gingivae and retromolar trigone behaved as an independent prognostic factor for late stage at diagnosis. These findings may well be explained by the fact that patient’s self-perception and self-exploration abilities depend on the site of the tumour (25), and also because gingival locations are associated to advanced stages at diagnosis (late diagnosis) due to the early invasion of the adjacent tissue (T4 primary tumour) (26).

Advanced-stage diagnosis in oral cancer has traditionally been attributed to delays in reaching a diagnosis, as patients at advance tumour stages are more likely to have longer patient and professional delays than those at early stages (27). However, the lack of sound scientific evidence supporting the existence of an association between diagnostic delay, extent of the disease (III-IV TNM stages) and lower survival rates is evident (12-14). This fact is probably related to a series of limitations and methodological flaws identified in the published reports to date, mainly related to heterogeneity in both the definition and measurement of diagnostic delay, the retrospective nature of these studies and also to a memory bias of the patients (12,13).

In this study, diagnostic delay was not significantly linked to advanced stage at diagnosis; thus the quickness in obtaining a diagnosis does not guarantee an early-stage tumour, although delay in the diagnosis of a neoplasm is universally considered unacceptable.

On the other hand, poor differentiation of the tumour (biologically more aggressive) behaved as an independent risk factor for diagnosis at stages III-IV. The tumour growth rate may play the role of a confounding factor in the relationship between diagnostic delay and disease-stage or survival, as patients with aggressive tumours and bad prognosis do not usually present diagnostic delay whereas tumours with low proliferation rates elicit good prognosis despite long diagnostic delays (28). Unfortunately, the evidence on tumour proliferation activity that could corroborate this hypothesis is scarce.

This paradoxical circumstance has previously been described in breast, cervix, lung, colon, renal, and urethral cancers and seems to suggest that stage at diagnosis is affected more by the biology of the cancer (rapid tumour growth) than by diagnostic delay (28,29). These results seem to suggest that the stage of oral cancer at the time of diagnosis is affected more by the biology of the cancer (degree of differentiation) than by diagnostic delay.

Taking into account that early diagnosis is a foremost step for reducing cancer mortality, it is concluded that the efforts aimed at early diagnosis of oral cancer should be prioritized towards screening programmes designed to detect the disease during its asymptomatic phases. Educational interventions on the population, particularly focused on risk groups (self-exploration) and on the professionals (clinician’s index of suspicion) should include a sound knowledge of the disease presentation, specifically on sites like floor of the mouth, gingivae and retromolar trigone. More studies are needed in order to analyse the part of tumour biology on the extension of the disease at the time of diagnosis.
